# A correlation study of music training, adult attachment, and personality traits using a large-sample questionnaire

**DOI:** 10.3389/fpsyg.2023.1218848

**Published:** 2023-08-24

**Authors:** Ying Liu, Xiaolin Liu, Maoping Zheng

**Affiliations:** ^1^School of Music, Southwest University, Chongqing, China; ^2^Mental Health Institute of Chinese Music, Chongqing, China

**Keywords:** music training, adult attachment, personality, large-sample questionnaire, correlation analysis

## Abstract

**Introduction:**

Music training can provide benefits to psychological health and is strongly associated with adult attachment style and personality traits through bidirectional interactions.

**Methods:**

A large sample including 10,191 Chinese musicians and non-musicians completed the Relationship Questionnaire and Big-Five Personality Inventory.

**Results:**

Connections between music training, adult attachment, and personality were analyzed with the following results: (1) personality traits were correlated with attachment dimensions, with positive correlations between secure attachment and extroversion and between dismissing attachment and neuroticism; (2) music training was connected with the secure and fearful-avoidant attachment dimensions, which complemented the effect of educational level in the preoccupied and dismissing dimensions; (3) music training enhanced extroversion, openness, agreeableness, and conscientiousness, which interacted to affect extroversion and openness by gender; and (4) music training enhanced the regression of extroversion to preoccupied and secure attachments, and the regression of openness to preoccupied attachment.

**Discussion:**

Conclusively, music training enhanced the connection between secure attachment and positive personality traits and regulated the correlation between insecure attachment and neuroticism. This analysis provides a large sample of statistical and practical evidence for the clinical practices of mental health and the educational and music training practices of co-cultivating cultural knowledge and artistic quality.

## 1. Introduction

The past few decades have seen increasing research on the psychological aspects of music using behavioral and neural measures (De Witte et al., 2020; McCrary et al., 2022). Even in daily music activities, music training affects attachment patterns and personality characteristics because of its psychological function in arousing/regulating emotion, facilitating cognition, memory recall, and activating sensorimotor functions (Corrigall et al., 2013; Haslam et al., 2022). Correspondingly, the influence of psychological traits on music training has also attracted attention in the contexts of human interaction and human–computer interaction (Penhune, 2020; Miranda, 2021; Tsai and Liang, 2023). With the increasing demand for social mental health services, the large-sample exploration of the relationship between music training and typical psychological characteristics will not only promote the clinical application of music to mental health but also help optimize the artistic activities related to music training, music appreciation, music creation, and so on.

### 1.1. Music and attachment

Music is a widely accepted medium for emotional expression and social communication. It has been used to promote attachment between parents and infants, treat children with abnormal emotional diseases, and improve negative attachment state in adults (Pasiali, 2014) because of its mechanism for enhancing social bonds, and regarding coalition signaling or the signaling of parental attention as a substitute for parental proximity (Cross, 2021; Savage et al., 2021). Attachment is an emotional reflection of the secure, positive, and stable emotional connections between individuals and intimate others (Bowlby and Ainsworth, 2013). This important psychological variable can predict the emotional response of individuals toward others and is most commonly categorized as secure attachment, preoccupied attachment, fearful-avoidant attachment, and dismissing attachment (Maalouf et al., 2022; Gori et al., 2023) classified by an avoidant dimension and anxious dimension (Liu et al., 2017). Individuals with secure attachment (low avoidance and low anxiety) typically have an internal sense of worthiness (lovability) and an expectation that other people are generally accepting and responsive. Individuals with preoccupied attachment (low avoidance and high anxiety) usually have a sense of unworthiness (unlovability) combined with a positive evaluation of others, which leads an individual to strive for self-acceptance by gaining the acceptance of valued others. Individuals with fearful-avoidant attachment (high avoidance and high anxiety) also have a sense of unworthiness coupled with an expectation that others will be negatively disposed, and avoid close involvement with others. Finally, dismissing attachment (high avoidance and low anxiety) indicates a sense of love-worthiness combined with a negative disposition toward others. This negative disposition acts to protect themselves against disappointment by maintaining a sense of independence and invulnerability (Bartholomew and Horowitz, 1991; Wongpakaran et al., 2021). The emotional impact of individual attachment style is closely related to individuals’ responses to music.

Music can increase the emotional connection of close relationships and reduce anxiety during emotional and non-emotional events. By reviewing attachment-focused music therapy literature, Stubbs (2018) proposed that the child-caregiver attachment relationship has been a focus of music therapy when caring for children hospitalized with life-threatening situations. An online questionnaire during the COVID-19 quarantine assessed the use of music in the home of young children and their parents. After controlling for relevant parent variables including parent distress, efficacy, education, and parent–child engagement in non-musical activities, the researchers found that parent–child musical engagement was strongly associated with parent–child attachment (Steinberg et al., 2021). The emotional value of music has also attracted much attention in cognitive neuroscience, revealing the overlapping neural characteristics between adult attachment and music-evoked emotion. The dopaminergic mesolimbic reward pathway is sensitive to both music-evoked pleasure (including the nucleus accumbens) and attachment-related emotions (Sescousse et al., 2013). During musical activity, the amygdala plays a prominent role in processing socio-affective significance and coding it with positive or negative emotional value (Koelsch, 2014). The hippocampal formation also participates in creating musical emotions related to social attachments (Frühholz et al., 2016). To a certain extent, the long-term emotional influence of music training can also affect the emotional connections between individuals and their intimate others, which not only affects their emotional responses but can also subtly change their attachment style (Luo et al., 2014; Kreutz and Feldhaus, 2020). Music intervention not only promotes romantic attachment and peer attachment (Chen-Jung et al., 2019) but also plays a positive role in the attachment behavior between parents and infants (Mmus, 2011). As a complex emotional pattern, attachment is interactively affected by gender (Kirkpatrick and Davis, 1994), educational level (Monteiro et al., 2007), personality trait (Carver, 2016), and many other variables. Consequently, to improve the application of music in the field of mental health, it is necessary to consider the interactive influence of personality and other factors while exploring the relationship between music and attachment.

### 1.2. Music and personality

Music training is not only associated with attachment styles within individuals but also with individual personality traits. Personality is an important psychological characteristic that reflects individual behavior tendencies with stability across time and space (Cobb-Clark and Schurer, 2012). Traits are commonly identified using the Big Five Personality Inventory across five dimensions: extroversion, agreeableness, conscientiousness, neuroticism, and openness (Gerber et al., 2011). Extroversion describes an individual’s energetic approach toward the social and material world. Agreeableness describes an individual’s prosocial and communal orientation toward others. Conscientiousness describes whether an individual has the socially prescribed impulse control that facilitates task- and goal-directed behavior. Neuroticism is considered to be the inverse trait of emotional stability. Openness describes an individual’s breadth, depth, originality, and complexity of mental and experiential life (Allbeck and Badler, 2002; Afolabi, 2013).

The dynamic two-way interaction between music training and personality has become the focus of psychological research in recent years. On the one hand, personality characteristics can affect individuals’ music preferences (Flannery and Woolhouse, 2021), music-induced movement or dancing style (Luck et al., 2010), and the durability and effectiveness of music training (Swaminathan and Schellenberg, 2018) across different ages (Luo et al., 2014). On the other hand, music training can improve the personality characteristics of individuals. Music training has been found to affect openness, conscientiousness, and neuroticism (Chamorro-Premuzic and Furnham, 2007) by improving cognitive processing, attention, perceptual integration, and memory processing (Miendlarzewska and Trost, 2014). In the relevant neural research, music training was found to affect personality development by shaping structural development (Hyde et al., 2009) and functional organization in the brains of children (Moreno et al., 2009). In turn, personality was found to modulate neural responses to musical emotions, with significant positive correlations between neuroticism scores and neural activity in the bilateral basal ganglia, insula, and orbitofrontal cortex (Park et al., 2013). When measuring electrodermal activity and the heart rate of listeners during shivering, a strong connection was also found between listeners’ music listening habits and personality, particularly that extroversion was negatively related to physiological reactions, while openness was positively related to physiological reactions (Starcke et al., 2019). Furthermore, personality and gender were found to predict music preference (Xu et al., 2021), and different music styles were associated with differing judgments of heterosexual attractiveness (Yang and Li, 2013), which could affect the romantic attachment of adults in short or long-term relationships.

The influence of music training on individuals not only affects emotional dimensions or personality features but creates an interaction correlation between multiple variables. Previous studies on the relationship between attachment and personality have found that individuals with secure attachment have higher scores in agreeableness and openness (Levine et al., 2021), while attachment anxiety correlates most strongly with borderline personality disorder (Smith and South, 2020). Alaei et al. (2022) found that individuals with higher attachment avoidance preferred the songs of close relationship with lyrics expressing an avoidant attachment style, whereas individuals higher in neuroticism preferred relationship songs with lyrics expressing attachment anxiety. However, evidence to describe the connections between music training, personality, and attachment, remains scant. To further explore the influence of music training on individual psychological traits and provide quantitative support for better attachment relationships and personality traits in those with music training, this study analyzed the relationship between music training, attachment styles, and personality traits from large-sample survey data. Based on the available evidence on the effects of music on adult attachment and personality traits, we hypothesized that (1) music training would be associated with the secure attachment style and would positively interact with all five personality traits; and that (2) music training would affect the correlation between attachment style and personality traits and would interact with other demographic characteristics.

## 2. Methods

### 2.1. Participants

This study was approved by the Institutional Reviewing Board of Southwest University, China. To clarify the relationship between music training, attachment, and personality, we conducted a large-sample online survey with Chinese adults, which included questionnaires on demographic characteristics, music training, the big five personality traits, and attachment style. Data were collected from residents distributed in central, east, north, southwest, and southeast China, including 27 universities and 23 cities. All data from the demographic survey, the Relationship Questionnaire, and the simplified NEO Five-Factor Inventory were anonymized and kept strictly confidential: this was overseen by the Academic Committee of the School of Music, Southwest University.

### 2.2. Measures

Demographic data were collected including gender, age, duration of music training, and educational level. Ultimately 10,191 valid responses (7,487 females and 2,704 males) from Chinese musicians and non-musicians were selected. The sample included 5,451 professional musicians (with over 7 years of professional music training) and 4,740 non-musicians (with no more than 1 year of professional music training). Regarding age, 8,866 participants were 18 ~ 25 years old, 1,030 participants were 26 ~ 45 years old, 251 participants were 45 ~ 60 years old, and 44 participants were 60 years old or above. Regarding education level, 156 participants were high school graduates, 8,886 participants had a bachelor’s degree, 1,063 participants had a master’s degree, and 146 participants had a doctoral degree (Table 1).

**Table 1:** The demographic details of participants.

Gender	Female	Male		
Number	7,487	2,704		
Age	18–25	26–45	46–60	60+
Number	8,866	1,030	251	44
Music training	Musicians	Non-musicians		
Number	5,451	4,740		
Educational level	High school	Bachelor’s degree	Master’s degree	Doctoral degree
Number	156	8,886	1,063	146

Adult attachment information was measured using the Relationship Questionnaire (RQ), which is based on Bartholomew and Horowitz’s four-category, two-dimensional model (Bartholomew and Horowitz, 1991). The RQ is a forced-choice instrument in which the four attachment styles (secure, preoccupied, fearful-avoidant, or dismissing attachment) are described in brief paragraphs. Each style is then described with a declarative sentence that allows participants to self-identify their attachment styles. The respondents rated the degree to which they resembled each style on a 5-point Likert scale from 1 (not at all applicable to me) to 5 (very much applicable to me). Lastly, a 4-point forced-choice item of attachment style was presented, and this response was used to categorize participants by attachment style. This questionnaire has adequate test–retest reliability and predictive validity (Ravitz et al., 2010; Wongpakaran et al., 2021), and it has been adopted globally.

The big five personality traits were assessed using the simplified NEO Five-Factor Inventory created by Costa and McCrae (1992) and translated by Chen and Xu (2015). It includes 60 items to measure extroversion, agreeableness, conscientiousness, openness, and neuroticism. All items were rated on a 5-point Likert scale from 1 (not at all applicable to me) to 5 (very much applicable to me). This questionnaire includes 32 forward-scoring and 28 reverse-scoring questions. The Cronbach’s alpha coefficient of the scale in this study was 0.93.

### 2.3. Statistical analyses

All analyses were conducted using IBM SPSS (IBM Corp., Armonk, NY, United States). Descriptive statistics were calculated to compare the effects of music training, gender, and educational level in attachment dimensions and personality traits. To examine attachment dimensions, we conducted three one-factor ANOVAs (including gender, music training, and educational level) on the self-identification scores across all four attachment dimensions. We then performed a MANOVA using these three factors to determine any interactions. To examine personality traits, we conducted three one-factor ANOVAs (including gender, music training, and educational level) on the self-identification scores across all five personality traits. We then performed a MANOVA using these three factors to determine any interactions. Partial correlations were used to analyze the correlation between attachment dimensions and personality traits while controlling for music training. A value greater than *r* = 0.3 (*p* < 0.01) was reported as a correlation and pictured by Matlab 2013. Linear regressions were calculated for the five personality traits to the four attachment dimensions within musicians and non-musicians, and for the four attachment dimensions to the five personality traits.

## 3. Results

### 3.1. Attachment dimensions

First, we conducted three one-factor ANOVAs of gender, music training, and educational level on the self-identification scores across all four attachment dimensions. Additionally, we conducted a MANOVA of the above three factors and only found a significant interaction for music training and educational level with dismissing attachment.

**Figure 1:**
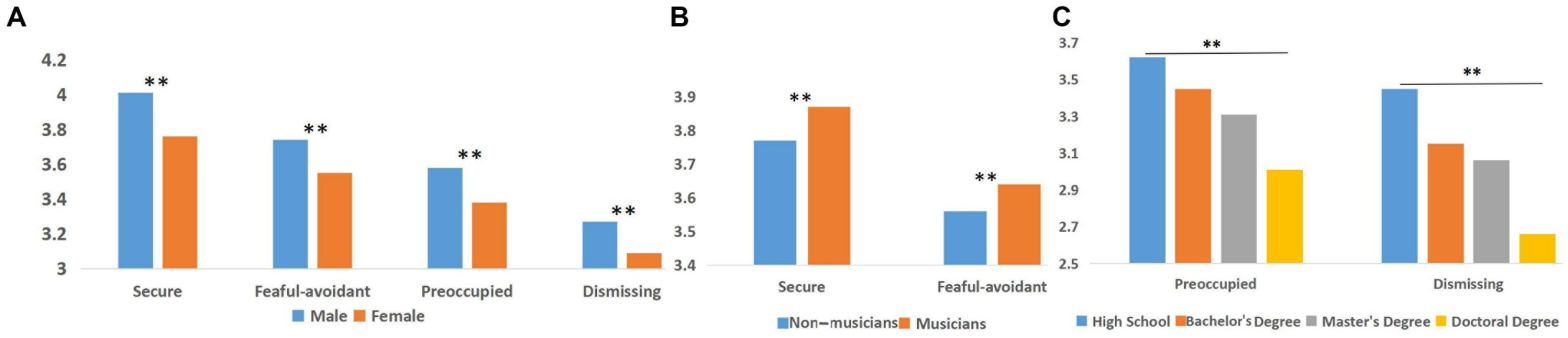
The Y axis is the score of attachment styles in RQ **(A)** Effect of gender on four attachment dimensions **(B)** Effect on music training on the secure and fearful-avoidant dimension **(C)** Effect of educational level on the preoccupied and dismissing dimensions.

**Figure 2:**
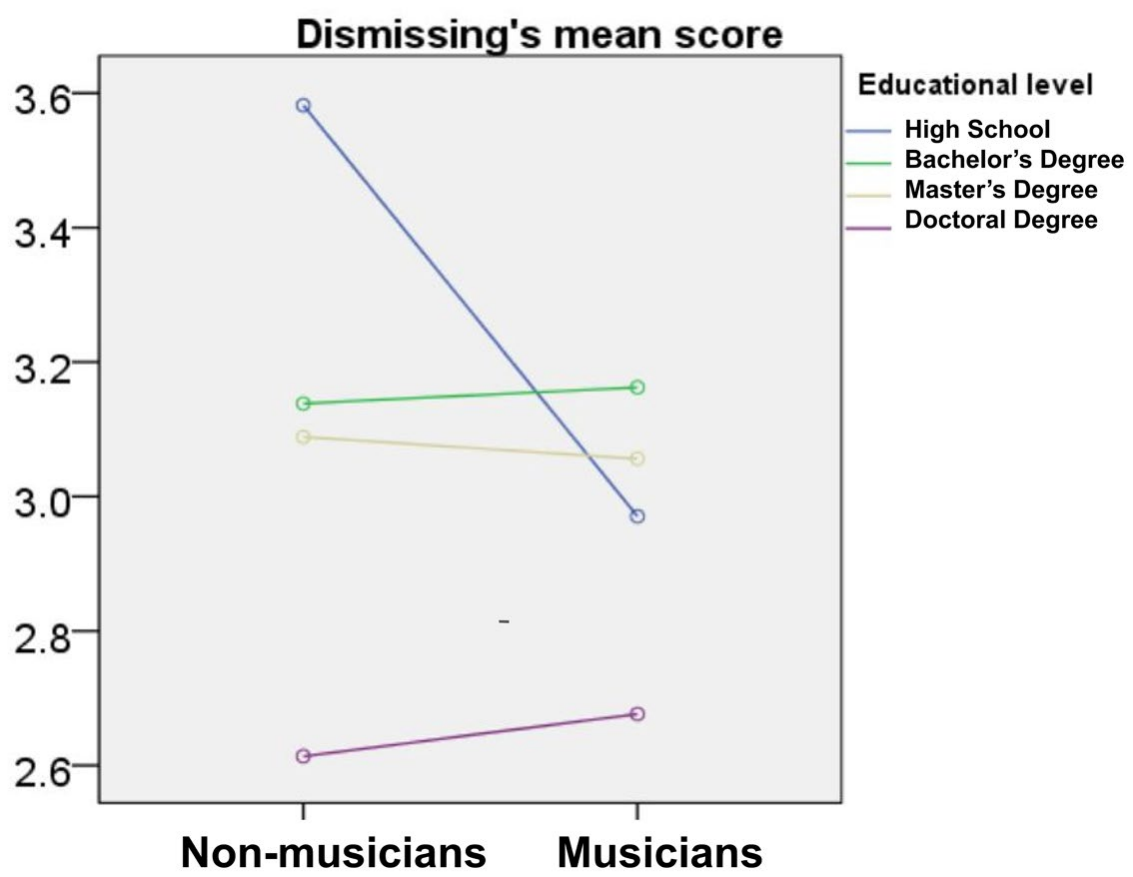
Scores on the dismissing dimension of musicians and non-musicians and across four educational levels.

A significant main effect of gender was found across all four attachment dimensions (Figure 1A): the attachment scores of males were significantly higher than those of females, *p* < 0.05. Significant main effects of music training were also found for secure attachment and fearful-avoidant attachment (Figure 1B). In the secure dimension, the mean score for musicians (3.87 ± 0.95) was higher than that for non-musicians (3.77 ± 0.96), *F* = 31.52, *p* < 0.01, *η* = 0.06. Similarly in the fearful-avoidant dimension, the mean score for musicians (3.64 ± 1.00) was higher than that for non-musicians (3.56 ± 1.00), *F* = 15.75, *p* < 0.01, *η* = 0.04. A main effect of educational level was found for the other two styles (preoccupied attachment and dismissing attachment; Figure 1C). In the preoccupied dimension, scores varied inversely with education level: the mean for participants with a high school degree (3.62 ± 1.33) was higher than the means for participants with a bachelor’s degree (3.45 ± 1.04), which was higher than the mean for those with a master’s degree (3.31 ± 1.08), which was higher than the mean for those with a doctoral degree (3.01 ± 1.21), *F* = 14.38, *p* < 0.01, *η* = 0.07. For dismissing style, a similar inverse relationship of mean scores was seen: high school (3.45 ± 1.33) > bachelor’s degree (3.15 ± 1.04) > master’s degree (3.06 ± 1.08) > doctoral degree (2.66 ± 1.21), *F* = 14.64, *p* < 0.01, *η* = 0.07. A significant interaction effect of music training and educational level was found for the dismissing style, *F* = 2.75, *p* < 0.05. A *post-hoc* analysis of the least significant difference showed that music training was associated with lower scores of those with dismissing attachment and a high school education more strongly than those with dismissing attachment and other educational levels (Figure 2).

### 3.2. Personality traits

Second, we conducted three one-factor ANOVAs of gender, music training, and educational level on the self-identification scores across all five personality traits. Additionally, we conducted a MANOVA of the above three factors and found a significant interaction of gender and music training in extroversion and openness and an interaction of gender and educational level in extroversion.

**Figure 3:**
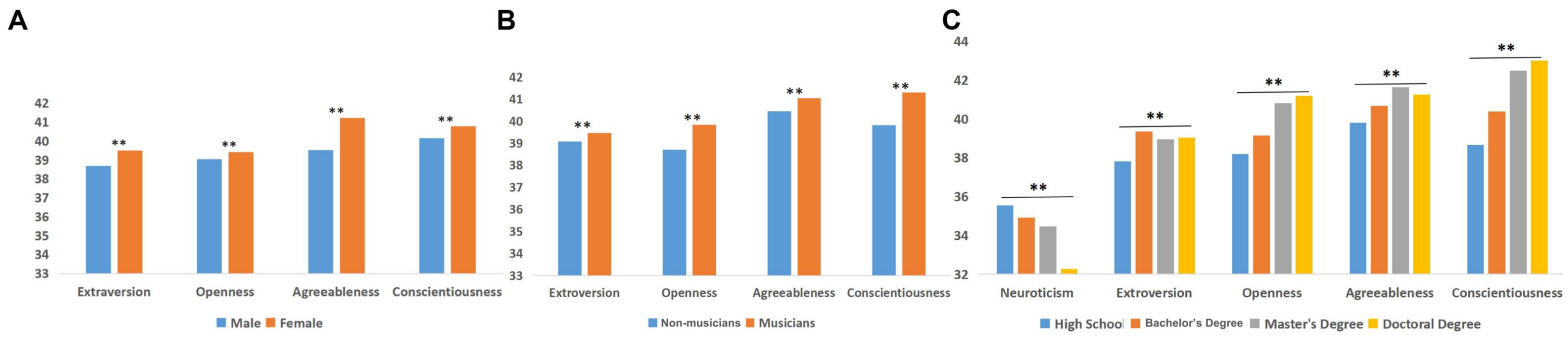
The Y axis is the score of personality traits **(A)** Effect of gender on personality **(B)** Effect of music training on personality **(C)** Effect of educational level on personality.

**Figure 4:**
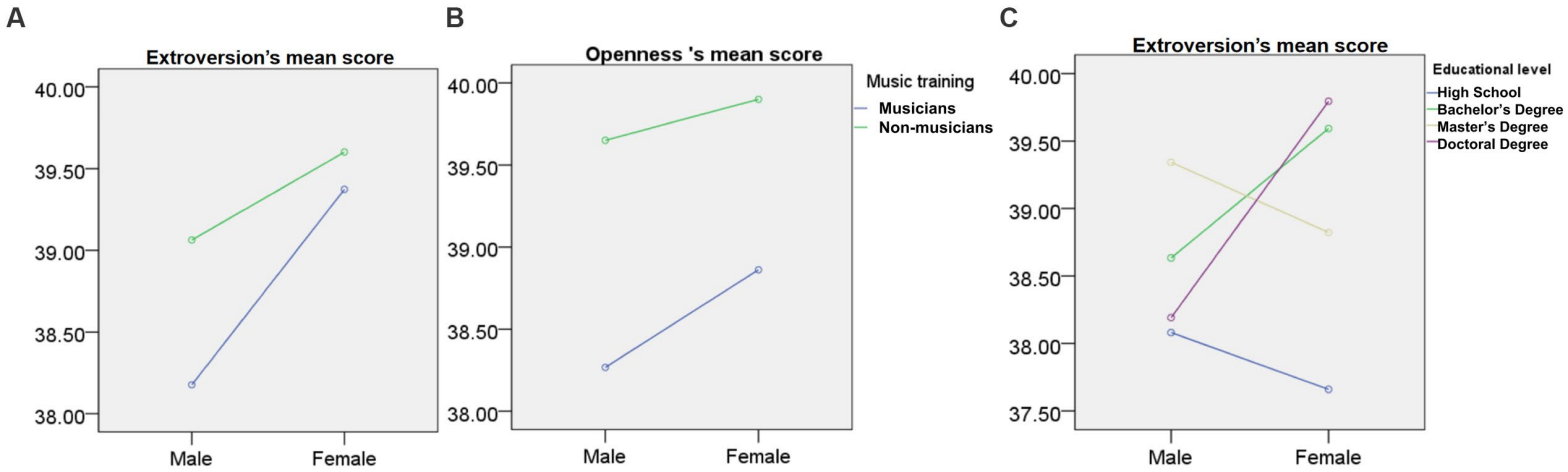
The Y axis is the score of personality traits **(A)** The interaction of gender and music training on extroversion **(B)** The interaction of gender and music training on openness **(C)** The interaction of gender and educational level on extroversion.

A main effect of gender was found for extroversion, openness, agreeable, and conscientiousness (but not for neuroticism): these scores were significantly higher in females than in males, *p* < 0.05 (Figure 3A). Similarly, a significant main effect of music training was found for the same four traits (Figure 3B). Musicians demonstrated significantly higher mean scores than non-musicians for the trait of openness, *F* = 209.48, *p* < 0.01, *η* = 0.14 (musicians [39.83 ± 4.03] > non-musicians [38.71 ± 3.72]); agreeableness, *F* = 35.99, *p* < 0.01, *η* = 0.06 (musicians [41.04 ± 5.06] > non-musicians [40.44 ± 4.90]); extroversion, *F* = 11.42, *p* < 0.01, *η* = 0.03 (musicians [39.45 ± 5.82] > non-musicians [39.07 ± 5.55]); and conscientiousness, *F* = 185.72, *p* < 0.01, *η* = 0.13 (musicians [41.30 ± 5.65] > non-musicians [39.82 ± 5.22]). Conversely, educational level had a significant main effect on all five personality traits (Figure 3C); specifically, the increased educational experience was associated with decreased neuroticism scores and increased scores of the other four personality traits. An interaction of gender and music training was found in extroversion and openness, *F*(extroversion) = 6.58, *p* < 0.05 (Figure 4A), *F*(openness) = 3.87, *p* < 0.05 (Figure 4B). An interaction of gender and educational level was found for extroversion, *F* = 4.59, *p* < 0.01 (Figure 4C).

### 3.3. Effects of attachment styles on personality traits

**Figure 5:**
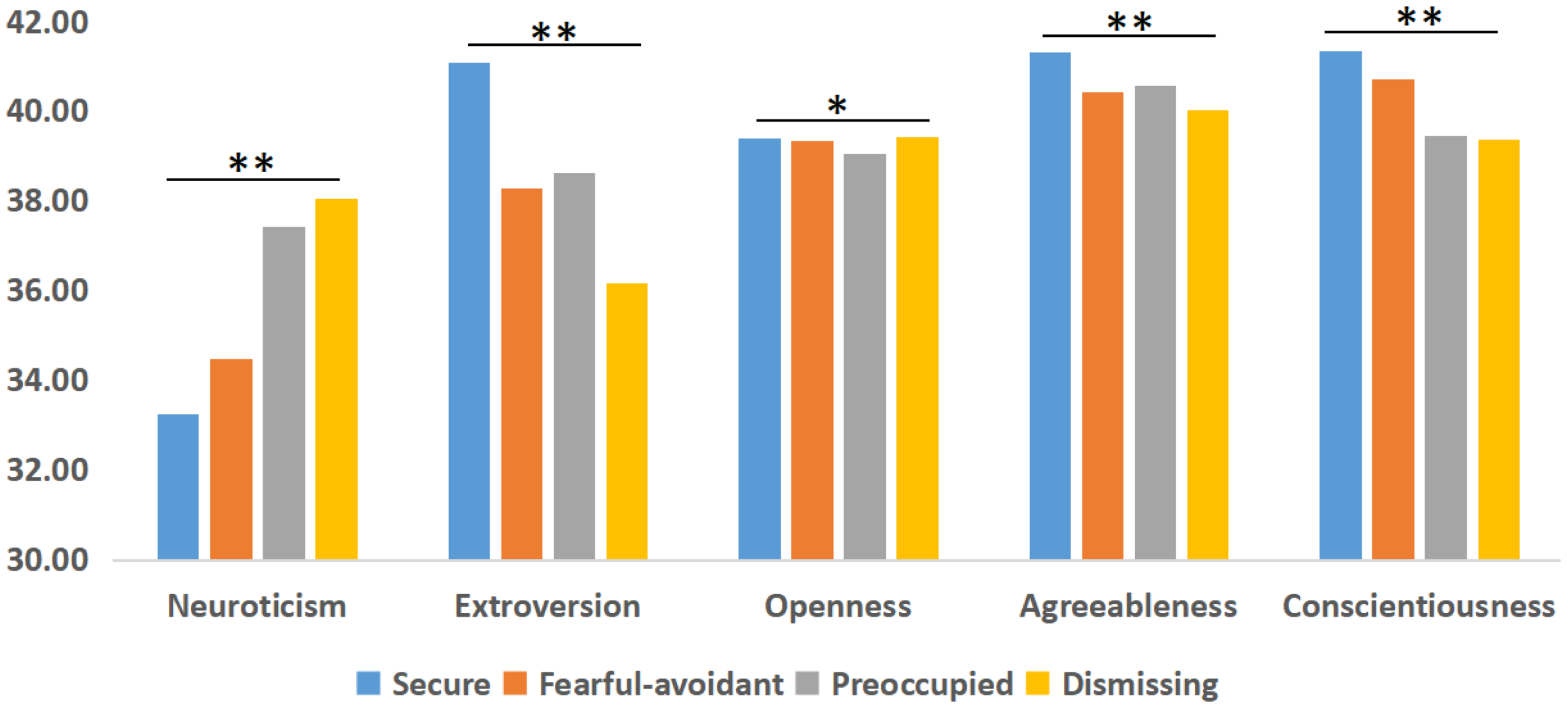
The mean scores of five personality traits among the four attachment styles.

A significant difference was found in the big five personality traits among the four attachment styles (Figure 5): *F*(neuroticism) = 257.19, *p* < 0.01, *η* = 0.27; *F*(extroversion) = 324.28, *p* < 0.01, *η* = 0.30; *F*(openness) = 3.48, *p* < 0.05, *η* = 0.03; *F*(agreeableness) = 30.86, *p* < 0.01, *η* = 0.10; *F*(conscientiousness) = 69.72, *p* < 0.01, *η* = 0.14.

### 3.4. Correlation analysis

**Figure 6:**
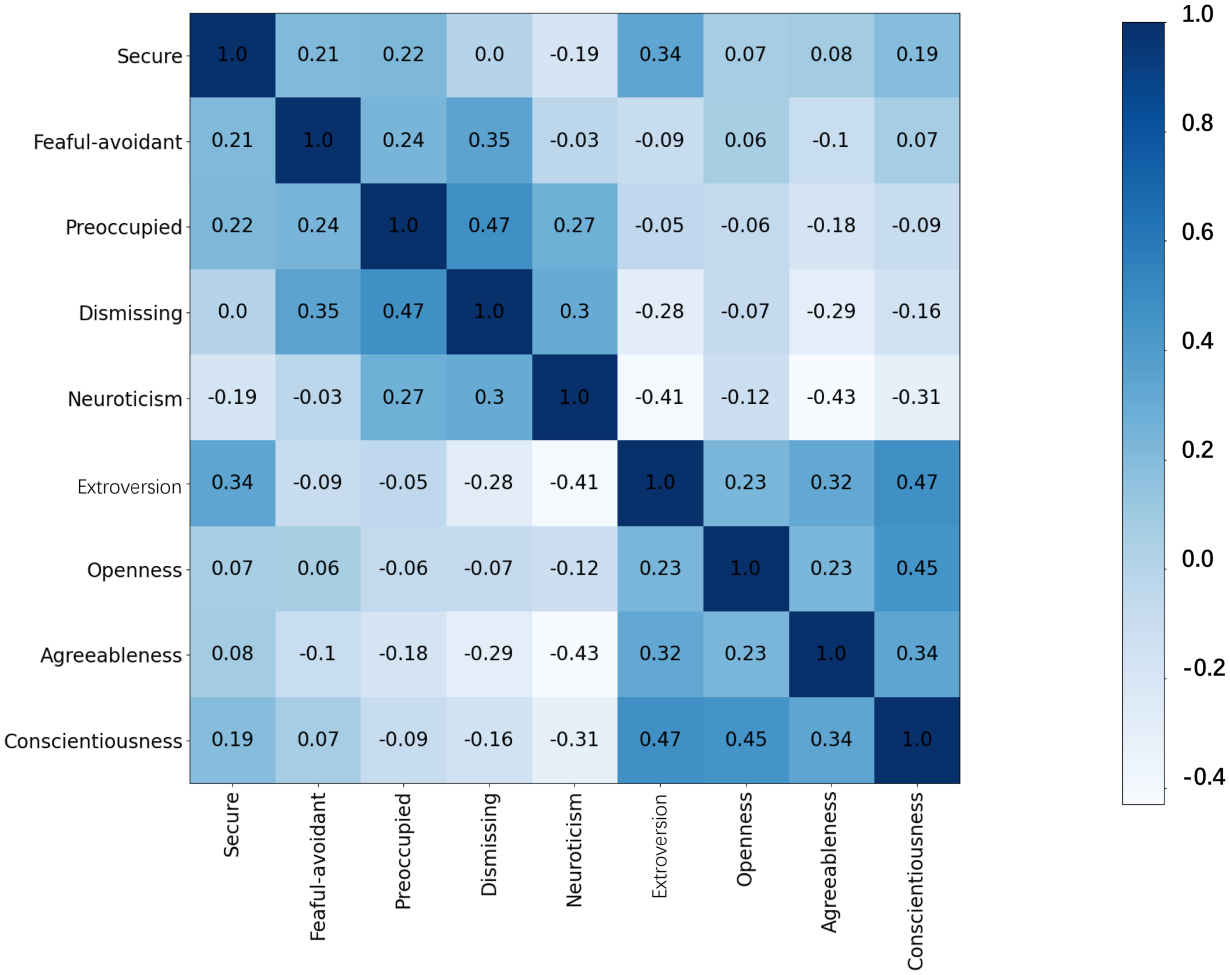
The correlation matrix of attachment dimensions and personality traits. The number in each box is the correlation coefficient.

The partial correlation of attachment dimensions and personality traits was analyzed with the control variable of music training. A value greater than *r* = 0.3 (*p* < 0.01) was reported as a correlation in the current study. In attachment dimensions, the dismissing dimension scores were correlated with the fearful-avoidant dimension (*r* = 0.35, *p* < 0.01) and the preoccupied dimension (*r* = 0.47, *p* < 0.01; Figure 6). For the big five personality traits, neuroticism scores were negatively correlated with scores for extroversion (*r* = −0.41, *p* < 0.01), agreeableness (*r* = −0.43, *p* < 0.01), and conscientiousness (*r* = −0.31, *p* < 0.01). Extroversion and agreeableness scores were positively correlated (*r* = 0.32, *p* < 0.01). Additionally, conscientiousness scores were positively correlated with extroversion (*r* = 0.47, *p* < 0.01), openness (*r* = 0.45, *p* < 0.01), and agreeableness (*r* = 0.34, *p* < 0.01). When comparing attachment dimensions to traits, significant positive correlations were found between the secure dimension and extroversion score (*r* = 0.34, *p* < 0.01), and between the dismissing dimension and neuroticism score (*r* = 0.30, *p* < 0.01).

### 3.5. Regression analysis

**Figure 7:**
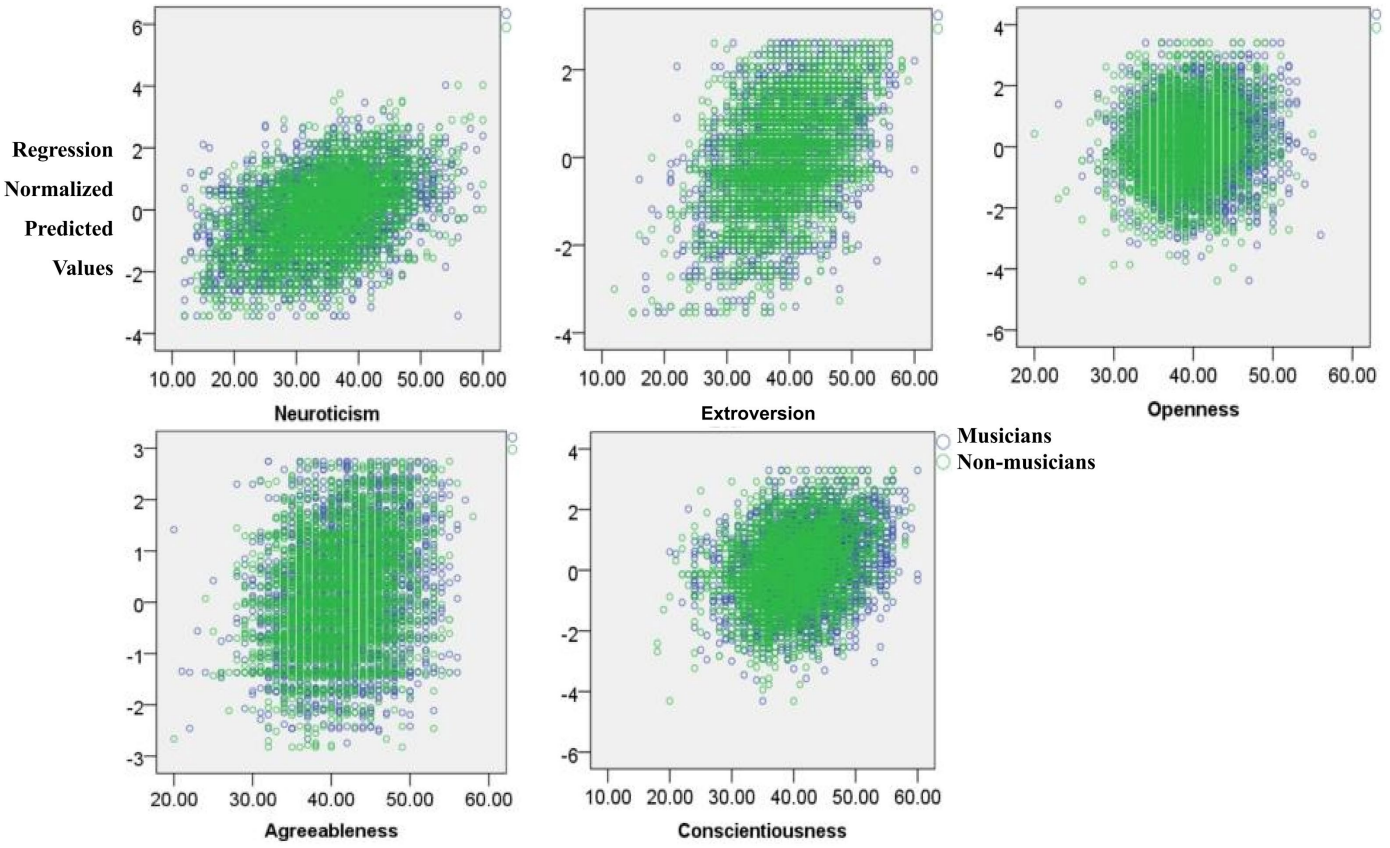
The scatter plots of the five personality traits regressed on attachment dimensions.

Lastly, to reflect the relationship between personality and attachment and how they interact with music training, a multiple regression analysis was performed on the original data. We calculated linear regressions within musicians and non-musicians of the five personality traits to the four attachment dimensions, and of the four attachment dimensions to the five personality traits because of the unknown interdependence between these factors (Figure 7). First, the personality traits were chosen as the dependent variable and the attachment dimension was chosen as the independent variable; these results are displayed in Table 2. Next, the inverse multiple regression analysis of the attachment dimensions to personality traits was performed using the original data, which was analyzed by the dependent variable of attachment dimensions and the independent variable of personality traits: these results are displayed in Table 3.

**Table 2:** Multiple regression results of personality traits on the four attachment dimensions.

		*R*	*R*^2^	*F*	*p* (Total)	*p* > 0.05 in the personality traits
Neuroticism	Musicians	0.41	0.17	281.75	0.00	
	Non-musicians	0.43	0.18	274.45	0.00	
Extroversion	Musicians	0.45	0.21	353.70	0.00	
	Non-musicians	0.44	0.19	281.35	0.00	*p* (preoccupied) > 0.05
Openness	Musicians	0.13	0.02	21.52	0.00	
	Non-musicians	0.15	0.02	25.32	0.00	
Agreeableness	Musicians	0.31	0.10	143.64	0.00	*p* (fearful-avoidant) > 0.05
	Non-musicians	0.32	0.10	131.36	0.00	*p* (fearful-avoidant) > 0.05
Conscientiousness	Musicians	0.26	0.07	97.59	0.00	
	Non-musicians	0.29	0.09	111.45	0.00	

**Table 3:** Multiple regression results of attachment dimensions on five personality traits.

		*R*	*R*^2^	*F*	*p* (Total)	*p* > 0.05 in the attachment dimension
Secure	Musicians	0.37	0.13	172.91	0.00	
	Non-musicians	0.32	0.14	118.85	0.00	*p* (openness) > 0.05
Fearful-avoidant	Musicians	0.20	0.04	61.85	0.00	
	Non-musicians	0.22	0.04	40.02	0.00	
Preoccupied	Musicians	0.30	0.09	104.88	0.00	*p* (openness) > 0.05 *p* (conscientiousness) > 0.05
	Non-musicians	0.28	0.08	86.62	0.00	*p* (openness) > 0.05
Dismissing	Musicians	0.39	0.07	192.95	0.00	
	Non-musicians	0.38	0.14	159.01	0.00	*p* (openness) > 0.05 *p* (conscientiousness) > 0.05

## 4. Discussion

### 4.1. Correlation between music training and adult attachment

Music training can affect secure attachment and fearful-avoidant attachment. In the current study, higher scores of the secure and fearful-avoidant dimensions were found in musicians than in non-musicians. Music has been found to modify an attachment style or relationship by regulating an individual’s negative internal working models of intimate others (Stubbs, 2018). Although music training is associated with some performance anxiety, it confers positive emotional regulation overall when producing harmonious sound and engaging in interpersonal interaction, benefits which may also be seen in music therapy. Previous studies found that family-centered music therapy facilitates secure attachment (Haslbeck and Hugoson, 2017) and decreases insecure attachment (Stubbs, 2018) through expressions of positive emotion and empathy. By systematically reviewing music interventions involving attachment relationships, Newman et al. (2022) found that music interventions could facilitate attachment-related changes by improving psychological processes such as increased parental sensitivity, reflective functioning, and emotional co-regulation. These studies all support the higher levels of secure attachment found in musicians in the current study.

However, musicians also demonstrated higher fearful-avoidant attachment scores than non-musicians in the current study. Individuals with fearful-avoidant attachment usually have a sense of unworthiness and avoid close involvement with others (Edelstein et al., 2012). The higher fearful-avoidant attachment style of musicians may represent a strategy of interpersonal avoidance to manage low-level mood swings and maintain a focus on professional development. Additionally, specific emotional stress associated with the long-term high-pressure learning process or performance anxiety of music training could explain this link (Oudejans et al., 2017; Fernholz et al., 2019). In many countries, music training is associated with the pressure of college entrance examinations and music skill grading tests (Rae and McCambridge, 2004; Zarza-Alzugaray et al., 2018; Zheng and Leung, 2021), which can easily enhance fear and avoidance in parent–child or peer attachments of musicians. Similarly, we found males to have higher fearful-avoidant attachment scores than females. Compared with females, males demonstrate a weaker mediating effect of cognitive reappraisals in emotional regulation (Zhang et al., 2020), which may result in less self-doubt and higher emotional stability in intimate relationships. When comparing the overall effect of gender on adult attachment, males showed significantly higher scores in the four attachment dimensions than females. Both these findings support the influence of music training and gender on fearful-avoidant attachment.

When comparing the effect of educational level on adult attachment, people with higher education showed significantly lower scores on the preoccupied and dismissing dimensions, which may indicate further that music training plays a compensatory role in the other two dimensions (secure and fearful-avoidant) or that emotional education may be necessary for those with a lower level of education. Music training was specifically associated with lower dismissing attachment scores only in participants with a high school education. This interaction of music training and educational level in the dismissing dimension indicates that increasing secure attachment through music training may be harder to achieve in those with lower education than in those with higher education. In other words, music training may be more necessary to help people with lower levels of general education to regulate their intimate relationships, which could show comparably or even better the improvement of music training in cognitive ability (Jaschke et al., 2013; Swaminathan and Schellenberg, 2020). In general, the connection between music training and the attachment dimensions seen in this study can not only be helpful to regulate one’s attachment style, but it can also be applied to improve educational philosophy and operational methods.

### 4.2. Correlation between music training and personality traits

In early studies, researchers could not define the relationship between musical ability and personality because of limitations within the sample variance. Since personality represents a relatively stable pattern of cognition, emotions, and behaviors, researchers have focused on personality to explore the relationship between music training and personality traits, both theoretically and practically (Miranda, 2019). Musicians were previously characterized by introversion, anxiety, intelligence, and good upbringing, as they were able to focus fully on technical music skills and simultaneously withdraw into an imaginative mental state (Kemp, 1981). However, researchers have since found that music training positively correlates with extroversion and openness, traits associated with aesthetic and creative interests (Greenberg et al., 2015; Thomas et al., 2016). When investigating differences in personality traits between professional musicians and the general workforce, Vaag et al. (2018) found that musicians displayed higher degrees of neuroticism and openness than the general workforce, as well as lower degrees of conscientiousness. Recently, a large-sample survey of personality differences (with more than 7,000 participants) found that musicians scored higher on neuroticism and lower on agreeableness and conscientiousness than amateurs (Kuckelkorn et al., 2021). However, in the current study, musicians showed significantly higher personality traits of extroversion, openness, agreeableness, and conscientiousness, but no significant difference was found for neuroticism.

The finding of higher extroversion and openness is consistent with previous studies, which supports the potential enhancement of music training on an individual’s prosocial attitude and broad motivations toward acceptance and learning (Nie et al., 2022). As an important social and emotional medium, music training was also found to interact with extroversion and openness by gender. In musicians, both males and females showed an enhanced tendency toward extroversion and openness. Corrigall et al. (2013) found that of the personality dimensions, openness had the best predictive power of the correlation between musical involvement and personality. A multivariate analysis of covariance showed significantly higher levels of extroversion in vocalists when compared to instrumentalists (Torrance and Bugos, 2017). Conjunctively, extroversion and openness can help the musicians to interact with external information (Costa-Giomi, 2012), which can help facilitate the positive effects of music training on personality building and enhance adaptation to abnormal environments.

The current result differed from the correlations of Vaag et al. (2018) and Kuckelkorn et al. (2021) of music training with agreeableness, conscientiousness, and neuroticism. In this study, positive correlations with extroversion were found with agreeableness and conscientiousness, which is consistent with other prior findings (Yu et al., 2021). Previous studies have identified a positive connection between well-being and extroversion, openness, agreeableness, and conscientiousness, as well as a positive connection between music training and well-being (MacDonald, 2013; Welch et al., 2020). Taken together, it is understandable that music training could enhance agreeableness and conscientiousness. For neuroticism, no significant difference was found between musicians and non-musicians, but a significant difference was found across people with different educational levels: as education level increased, neuroticism scores decreased, whereas the scores of the other four personality traits showed an upward trend. Miranda (2020) proposed that neuroticism is a universal aspect of personality that affects musicians’ musical behaviors by providing greater insight into musical emotions, musical habits, and music making. The absence of the relationship between music training and neuroticism here could indicate the limitation of the questionnaires used. Because of its connection with emotional instability, this absence could also suggest the positive emotional effects of general education to decrease neuroticism.

Other results indicated that cultural differences might be considered as a source of variance between music training and personality (Kotsopoulou and Hallam, 2010; Lima et al., 2020). The participants in this study were Chinese, who differ widely from Western populations in personality traits (Cheung, 2004), music preference, aesthetic bias, and the social value of music (Garrison, 2012; Tan and Min, 2021). Overall, these results provide evidence to describe the relationship between music training and several personality traits, and provide a potential educational application of music training.

### 4.3. The interactive relationship between music training, attachment, and personality

Revealing the interaction of music training with attachment and personality may provide comprehensive evidence of emotional and behavioral characteristics in the feature analysis and practical applications of music training. Significant correlations were found within and between the attachment dimensions and personality traits (Figure 5). Primarily, the correlation between attachment dimensions and personality traits found that neuroticism was positively associated with dismissing attachment. Neuroticism is accompanied by a pervasive perception that the world is a dangerous and threatening place and is often accompanied by beliefs about one’s inability to manage or cope with challenging events (Barlow et al., 2014). Among the five personality traits, neuroticism showed a negative correlation with the other four traits, while conscientiousness showed a positive correlation with the other traits. Similarly, the trends of traits across the four attachment dimensions showed an opposite pattern for neuroticism: the lowest score was in the secure dimension. Within the four attachment dimensions, the dismissing dimension also showed a significantly positive correlation with the fearful-avoidant and preoccupied dimensions, which is consistent with an internal working model of dismissing attachment in which hyperactivating reactions are experienced as delusions, hallucinations, or suspiciousness (Dozier and Lee, 1995; Meyer and Pilkonis, 2001). Combined with Miranda’s (2020) opinion that neuroticism can help people to gain greater insight into musical emotions and processes, we conclude that neuroticism can help musicians experience emotional information in music training, but it does not enhance their security within intimate relationships. A significant positive correlation was also found between extroversion and secure attachment. The construction of secure intimate relationships may require individuals to maintain an openness and extroversion (Green-Hennessy and Reis, 1998; Kwon and Choi, 2022). A strong connection may exist between attachment and personality, particularly when integrating one’s internal emotional attitude and interpersonal beliefs into the external environment.

When comparing the role of music training in attachment and personality using a multiple regression analysis, a significant difference was found between musicians and non-musicians, indicating the influence of music training on the relationship between attachment and personality. Extroversion was significantly regressed in the preoccupied dimension of musicians but not of non-musicians (Table 1), which implies that music training could enhance the relationship between extroversion and preoccupied attachment. When the regression variable was exchanged (Table 2), the secure and preoccupied dimensions were significantly regressed for openness in musicians, but not in non-musicians. Along with low avoidance and high anxiety, individuals with preoccupied attachment shared a low defensive belief with those with secure attachment; low defensiveness is consistent with the emotional and cognitive basis of openness. Although preoccupied attachment is described as a sense of unworthiness combined with a positive evaluation of others (West and George, 2002), the regression findings here may indicate the positive regulative role of music training in enhancing openness within preoccupied individuals to help them strive for self-acceptance by gaining the acceptance of valued others through musical excellence or achievement.

### 4.4. Limitations

Using a large-sample survey, this research provided an effective description of relationships across music training, adult attachment, and personality. However, it has several shortcomings regarding the classification of participants and the application of neural methods. First, we did not compare differences across various types of music training, which may affect the connection of music training with personality traits and adult attachment. The aspect of musical style or instrument (including vocalists) should be subdivided in future research. Second, future studies should conduct cross-cultural research to investigate the differences of participants from different cultures, further enriching the investigation into the psychological characteristics of music training and the practical applications of the resulting evidence. Third, future research may also study electrophysiological signals to clarify the neural connection between different music training and individual emotional attachment style and personality traits, further explaining the behavioral and physiological influences of music. Finally, future research may also analyze this relationship using a formal mediation or moderation model.

## 5. Conclusion

The current study provides evidence of the correlations between music training, adult attachment, and personality. Through this large-sample survey, music training was found to enhance the connection between secure attachment and positive personality traits, as well as to regulate the correlation between insecure attachment and neuroticism. Additionally, music training can affect attachment and personality cooperatively with gender and educational level. As the first correlative study to examine music training, adult attachment, and personality, our findings can guide the clinical practices of mental health and the educational practices of music training, which co-cultivates cultural knowledge and artistic quality. Future studies should further explore the correlations between music training, adult attachment, and personality using more specific behavioral and neural experiments.

## Data availability statement

The raw data supporting the conclusions of this article will be made available by the authors, without undue reservation, after the completion of the related project.

## Ethics statement

The studies involving humans were approved by the Academic Committee of the School of Music, Southwest University. The studies were conducted in accordance with the local legislation and institutional requirements. The participants provided their written informed consent to participate in this study. The animal study was approved by the Academic Committee of the School of Music, Southwest University. The study was conducted in accordance with the local legislation and institutional requirements. Written informed consent was obtained from the individual(s) for the publication of any potentially identifiable images or data included in this article.

## Author contributions

All authors listed have made a substantial, direct, and intellectual contribution to the work and approved it for publication.

## Funding

This research was supported by the National Natural Science Foundation of China (32200823), the Fundamental Research Funds for the Central Universities of China (SWU2209508), the Chongqing Social Science Planning Project (2021BS104), and the Chongqing Graduate Education Reform Project (YJG233025).

## Conflict of interest

The authors declare that the research was conducted in the absence of any commercial or financial relationships that could be construed as a potential conflict of interest.

## Publisher’s note

All claims expressed in this article are solely those of the authors and do not necessarily represent those of their affiliated organizations, or those of the publisher, the editors and the reviewers. Any product that may be evaluated in this article, or claim that may be made by its manufacturer, is not guaranteed or endorsed by the publisher.
